# Increased P450 aromatase levels in post-menopausal women after acute ischemic stroke

**DOI:** 10.1186/s13293-020-00357-w

**Published:** 2021-01-07

**Authors:** Bharti Manwani, Pamela Fall, Liang Zhu, Meaghan Roy O’Reilly, Sarah Conway, Ilene Staff, Louise D. McCullough

**Affiliations:** 1 Department of Neurology and Neuroscience, University of Texas, Houston, TX USA; 2 University of Connecticut Health Center, Farmington, CT USA; 3 Department of Internal Medicine, University of Texas, Houston, TX USA; 4 Department of Neurology, Brigham and Women’s Hospital, Boston, MA USA; 5 Department of Research, Hartford Hospital, Hartford, CT USA

**Keywords:** Aromatase, Estradiol, Testosterone, Stroke, Sex differences

## Abstract

**Background:**

Sex differences in stroke have been attributed to the neuroprotective effects of estrogen, yet most clinical trials of estrogen supplementation for stroke prevention have failed. The contribution of sex hormones to stroke outcome remains a subject of debate. Aromatization of testosterone to estradiol in neural tissue leads to sexual differentiation. Emerging data suggests aromatase activity increases in response to brain injury, and increased aromatase expression is seen in the ischemic penumbra in animal models. The objective of this study was to examine the levels of endogenous sex steroids after acute ischemic stroke and determine if levels of sex steroids were associated with acute stroke outcomes.

**Methods:**

Peripheral blood from ischemic stroke patients and controls was collected under an approved IRB within 24 h of symptom onset. 17β-estradiol, testosterone, and aromatase levels were measured in the serum of both men and women using ELISA. Hormone levels were compared in men vs. women in stroke and control groups and correlated with outcomes (NIHSS and change in the modified Rankin Scale (mRS), defined as the difference of premorbid and discharge mRS) using multivariate regression.

**Results:**

We found no significant difference in estradiol levels 24 h after stroke in men (*p* = 0.86) or women (*p* = 0.10). In men, testosterone significantly decreased after stroke as compared with controls (1.83 ± 0.12 vs. 2.86 ± 0.65, *p* = 0.01). Aromatase levels were significantly increased in women after stroke as compared with controls (2.27 ± 0.22 vs. 0.97 ± 0.22, *p* = 0.002), but not in men (*p* = 0.84). Estradiol levels positively correlated with change in mRS in both women (*r* = 0.38, *p* = 0.02) and men (*r* = 0.3, *p* = 0.04).

**Conclusions:**

Estradiol levels correlated with functional outcomes (change in mRS) in both men and women, at least in the acute phase (24 h) of stroke. However, no significant difference in estradiol levels is seen 24 h post-stroke in men or women. Testosterone levels decrease at 24 h after stroke in men. As seen in animal models, aromatase levels increase after acute ischemic stroke, but this was only true for women. These indicate an active aromatization process in post-menopausal women after acute ischemic stroke.

## Introduction

It is well established that sex differences exist in stroke incidence, prevalence, and outcome in ischemic stroke [1]. Elderly women bear the major brunt of stroke disability as compared to men [2, 3]. Most of this sexual dichotomy in stroke has been attributed to the effects of sex hormones [4], as preclinical data has consistently shown that estrogens are neuroprotective [5]. However, clinical trials of estrogen supplementation have failed. In fact, the Women’s Health Initiative (WHI) and Women’s Estrogen for Stroke Trial (WEST) trials showed increased mortality in elderly women supplemented with estrogen [6, 7], although there were several issues with the trial design including enrollment of older women many years past menopause [8].

P450 aromatase is the enzyme that actively converts testosterone to estradiol in the embryonic brain. During prenatal development, aromatization of testosterone to estradiol in neural tissue leads to sexual differentiation/defeminization of the male brain [9, 10]. On the other hand, the female brain develops in the absence of estradiol as it is bound to α-fetoprotein, a plasma protein in the periphery [10]. Interestingly, increased aromatase expression is seen in the ischemic penumbra in animals [11]. Administration of aromatase inhibitors or deletion of aromatase increased ischemic damage in experimental stroke models beyond what was seen in ovariectomized mice, suggesting that endogenous extragonadal estradiol production is important in females for neuroprotection [12]. However, there are limited studies examining sex steroid levels in humans after ischemic stroke. The aim of this study was to assess the post-stroke sex steroid milieu in men and women. Previous studies have focused on either estradiol or testosterone levels in either men or women after stroke. Since there may be a complex dynamics of estradiol, aromatase, and testosterone level after stroke, we assessed the levels of these hormones simultaneously in both men and women.

## Methods

This study was conducted at Hartford Hospital, CT, a regional tertiary care facility with a Joint Commission certification as a comprehensive stroke center. Serum from patients presenting with focal neurological deficits who consent under an IRB-approved protocol to participate in a biobank study was collected within 24 h from symptom onset. Serum samples from 102 patients (61 men and 41 women, with 43 men and 32 women in the stroke group and 18 men and 9 women in the control group) were used for this study. Blood samples from patients were collected in vacutainer tubes, allowed to clot for 30 min, and then centrifuged to obtain serum, as described previously [13]. Serum was stored at − 80 °C until analysis and each sample was run in duplicates. A blinded investigator performed serum enzyme-linked immunoassay (ELISA) using kits for testosterone (Calbiotech, Spring Valley, CA, USA), 17β-estradiol (BQ, San Diego, CA, USA), and P450 aromatase (Cloud-Clone Corp, Houston, TX, USA), following the manufacturer’s protocol. The sensitivity of these ELISA kits was 0.1 ng/ml for testosterone, 10 pg/ml for 17β-estradiol, and 0.054 ng/ml for aromatase. The intra-assay and inter-assay coefficient of variability was < 10% and < 12%, respectively.

Patients were divided into ischemic stroke and control groups. Stroke was defined as an acute-onset focal neurological deficit with confirmation by radiographic imaging (CT or MRI). Patients who presented with focal neurological deficits with subsequent resolution of symptoms and no evidence of ischemia on CT/MRI were included in the control group (most were patients with a diagnosis of transient ischemic attack, seizures, complex migraines, or hypertensive encephalopathy). Exclusion criteria were age < 56 years, hemorrhagic stroke, any malignancy, autoimmune disease, immunosuppressive, hormone replacement therapy (testosterone replacement or steroid therapy including the use of oral contraceptives). Demographic data including past medical history was collected from the stroke patient database. Outcome measures were the National Institutes of Health Stroke Scale (NIHSS; range, 0 to 42, with higher scores indicating a greater deficit) at discharge and modified Rankin Scale (mRS; range of 0 [no symptoms] to 6 [death]). Premorbid (baseline mRS) and mRS at the time of hospital discharge were collected by patient chart review. Change in mRS (discharge − premorbid) was used for analysis [14].

SAS and graphpad PRISM software was used for data analysis. Two-way ANOVA was used to compare hormone levels between stroke/control and women/men groups. Post hoc subgroup comparisons within women and men were provided and adjusted for multiple testing by the Sidak method. Spearman correlation coefficient was calculated for hormone levels with NIHSS at the time of discharge and change in mRS (discharge − premorbid mRS) [14]. We performed univariate analysis for age, NIHSS, smoking, diabetes, and history of cancer on each hormone. Variables with a *p* value less than 0.1 in the univariate analyses were included in a multivariate linear regression to control for confounders. The criterion of statistical significance in the final model was set at 0.05.

## Results

Baseline characteristics of our study population are shown in Tables 1 and 2. There was no statistically significant difference in comorbidities including hypertension, hyperlipidemia, diabetes mellitus type 2, and atrial fibrillation in stroke vs. controls. Table 2 shows the stroke cohort demographics and outcomes. Women were older (79.2 ± 10.4 years) at the time of stroke as compared to men (72.8 ± 10 years), as described previously [15]. Women had higher median discharge NIHSS (men 2 ± 3 vs. women 3 ± 9, *p* = 0.032) and change in mRS (calculated as discharge − premorbid mRS) (men 2 ± 3 vs. women 4 ± 2, *p* = 0.009), indicating worse outcomes, which has been seen in previous studies [16].

**Table 1:** Patient demographics for stroke, *n* = 75 vs. controls, *n* = 27: There was no significant difference in premorbid conditions including Hypertension, Hyperlipidemia, Type 2 Diabetes Mellitus, Atrial fibrillation, or Smoking in stroke versus controls

Baseline characteristics	Stroke	Controls	***p*** value
Hypertension, *n* (%)	57 (76.0%)	20 (74.1%)	0.842
Hyperlipidemia, *n* (%)	46 (61.3%)	14 (51.9%)	0.391
Heart disease, *n* (%)	29 (38.7%)	8 (29.6%)	0.402
Type 2 diabetes mellitus, *n* (%)	23 (30.7%)	7 (25.9%)	0.643
Atrial fibrillation, *n* (%)	23 (30.7%)	5 (18.5%)	0.316
Smoking, *n* (%)	14 (18.7%)	2 (7.4%)	0.225

**Table 2:** Patient demographics for men and women with stroke: *n* = 32 women, *n* = 43 men. Women were significantly older at the time of stroke as compared to men, *p* = 0.016. There was no significant difference in premorbid conditions including Hypertension, Hyperlipidemia, Type 2 Diabetes Mellitus, Atrial fibrillation, or Smoking in men vs. women with stroke. Men had significantly higher heart disease history as compared to women, *p* = 0.036 in our patient population. Women had significantly worse outcomes as compared to men, in terms of discharge NIHSS, *p* = 0.032, and change in mRS, *p* = 0.009

Stroke category and outcomes	Men (***n*** = 43)	Women (***n*** = 32)	***p*** value
Age (mean ± SD)	72.8 ± 10	79.2 ± 10.4	0.016
Hypertension, *n* (%)	33 (76.7%)	24 (75.0%)	0.861
Hyperlipidemia, *n* (%)	29 (67.4%)	17 (53.1%)	0.208
Heart disease, *n* (%)	21 (48.8%)	8 (25.0%)	0.036
Type 2 diabetes mellitus, *n* (%)	12 (27.9%)	11 (34.4%)	0.548
Atrial fibrillation, *n* (%)	11 (25.6%)	12 (37.5%)	0.268
Smoking, *n* (%)	11 (25.6%)	3 (9.4%)	0.132
NIHSS on admission, median (IQR)	7(12)	13(18)	0.144
NIHSS at discharge, median (IQR)	2(3)	3(9)	0.032
Cardioembolic, *n* (%)	21 (48.8%)	22 (68.8%)	0.085
Large artery atherosclerosis, *n* (%)	7 (16.3%)	4 (12.5%)	0.749
Small vessel disease, *n* (%)	6 (14.0%)	3 (9.4%)	0.724
Undetermined etiology, *n* (%)	9 (20.9%)	3 (9.4%)	0.177
Baseline modified Rankin score, median (IQR)	0 (1)	0 (3)	0.084
Change in modified Rankin score, median (IQR) [discharge mRS − premorbid mRS]	2 (3)	4 (2)	0.009
Mortality	5 (11.63%)	5 (15.63%)	0.736

**Fig. 1:**
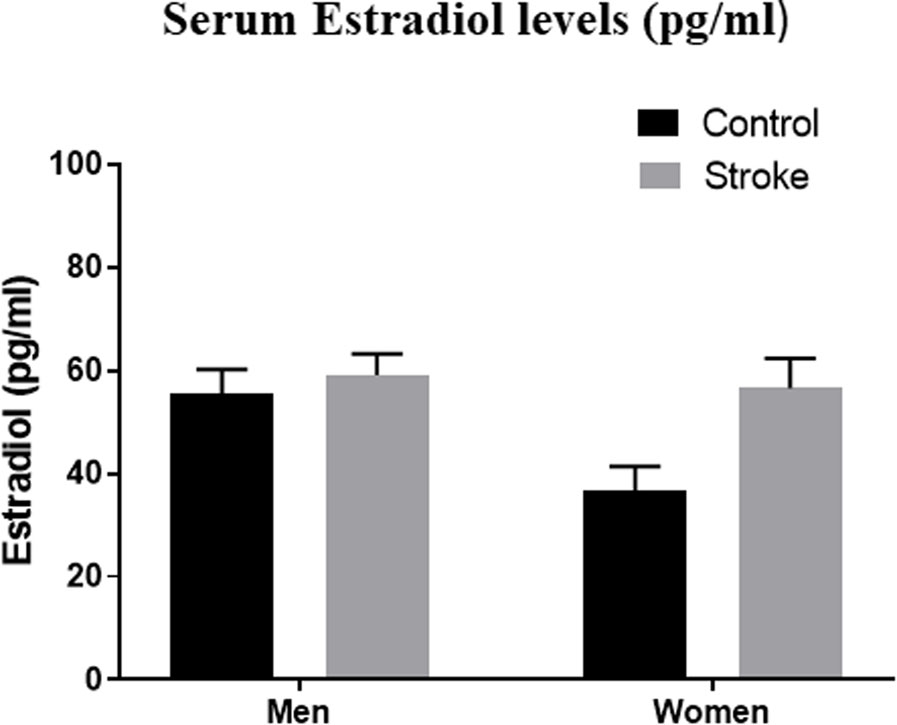
Serum estradiol levels in picograms per milliliter (pg/ml). There was no significant difference in estradiol levels 24 h post-stroke in men or women. In women, the estradiol levels trended up after stroke but did not reach significance, *p* > 0.5

We first compared levels of sex hormones in stroke patients with controls. There was no significant difference in estradiol levels after stroke in men (*p* = 0.86) or women (*p* = 0.10) (Fig. 1).

**Fig. 2:**
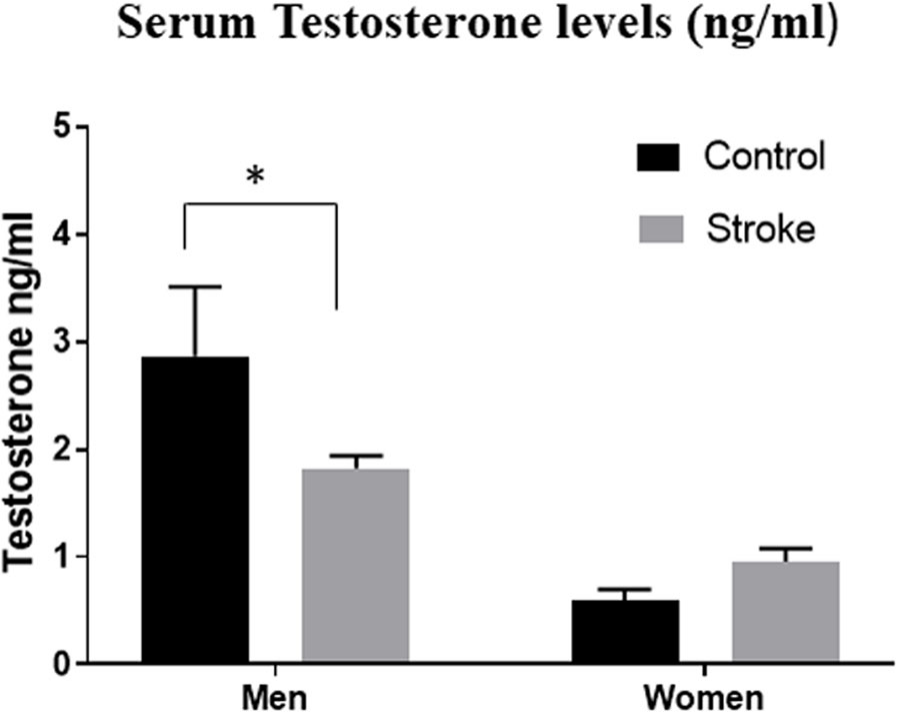
Serum Testosterone levels in nanograms per milliliter (ng/ml). The serum testosterone levels significantly decreased in men 24 h post-stroke. *p = 0.01

In men, testosterone significantly decreased after stroke as compared with controls (1.83 ± 0.12 vs. 2.86 ± 0.65, *p* = 0.01). No difference in testosterone levels was seen in women after stroke (*p* = 0.71) (Fig. 2). There was a significant main effect of sex (*F* (1, 98) = 26.27, *p* < 0.0001) and sex by stroke interaction (*F* (1, 98) = 5.24, *p* = 0.02).

**Fig. 3:**
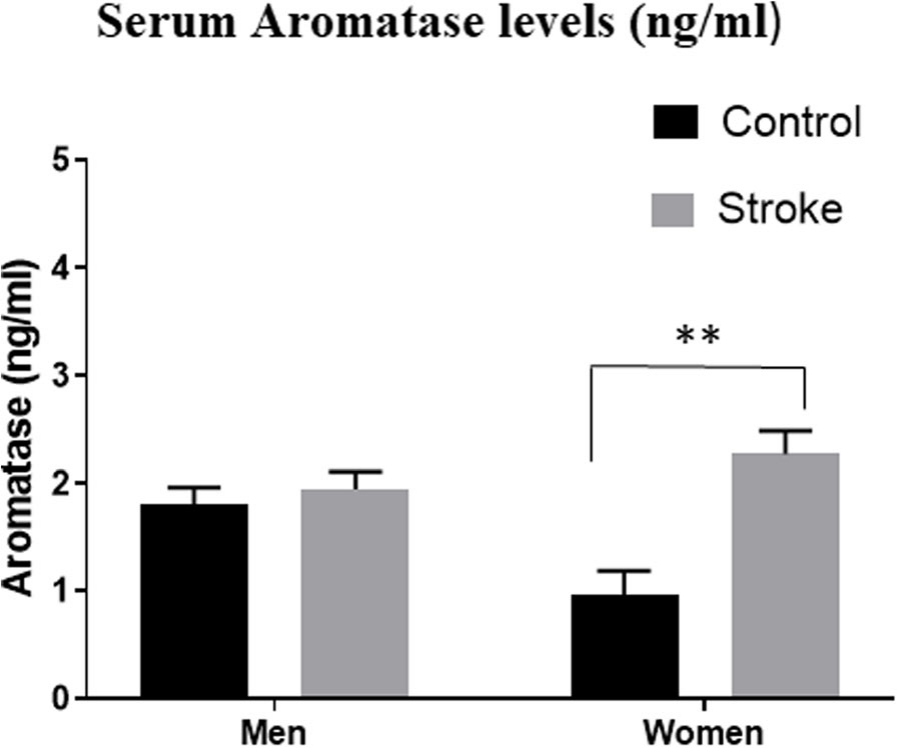
Serum Aromatase levels in nanograms per milliliter (ng/ml). The serum aromatase levels significantly increased in women after stroke. No such difference was seen in men. **p = 0.002

Aromatase levels were significantly increased in women after stroke as compared with controls (2.27 ± 0.22 vs. 0.97 ± 0.22, *p* = 0.002), but not in men (*p* = 0.84) (Fig. 3). For aromatase, there was a significant main effect of stroke (*F* (1, 98) = 9.09, *p* < 0.003) and sex by stroke interaction (*F* (1, 98) = 5.72, *p* < 0.01).

In women, there was a significant positive correlation between estradiol levels and NIHSS at the time of discharge, *r* = 0.45, *p* = 0.01. Similarly, estradiol levels positively correlated with change in mRS score, *r* = 0.38, *p* = 0.02, in women. This correlation was also seen in change in mRS, *r* = 0.3, *p* = 0.04, in men, but not in discharge NIHSS, *r* = 0.26, *p* = 0.1. Testosterone levels had a positive correlation with discharge NIHSS in women, *r* = 0.44, *p* = 0.01. There was no significant correlation of testosterone level with NIHSS in men or with change in mRS score in men or women, *p* > 0.05.

No significant correlation was seen between aromatase and NIHSS (men, *r* = − 0.3, *p* = 0.06; women, *r* = 0.3, *p* = 0.09) or change in mRS score (women, *r* = − 0.07, *p* = 0.6; men, *r* = − 0.2, *p* = 0.1). We used a multivariate regression model and controlled for factors like diabetes mellitus, hypertension, hyperlipidemia, age, and NIHSS at time of admission, and serum levels of estradiol were still independently associated with mRS in men and women.

## Discussion

Our study shows that aromatase levels are significantly increased in post-menopausal women after acute ischemic stroke when measured within 24 h from symptom onset. Testosterone levels significantly decrease in men at this time point. No significant difference in estradiol levels was seen at 24 h post-ischemic stroke in either sex, although there was a trend for increased estradiol levels in women with stroke. Interestingly, there was a significant positive correlation between estradiol levels and change in mRS (functional outcome measure), regardless of sex.

In intrauterine life, sexual differentiation of the brain occurs by aromatization of androgens to estrogens [9]. It has been shown that neurotoxic insult to the brain (induced by systemic administration of kainic acid) leads to increased aromatase expression in astrocytes [17]. In spontaneously hypertensive rats, aromatase was elevated at 24 h and at 8 days after focal cerebral ischemia in the penumbra, specifically in the astrocytic processes [11]. The expression of aromatase has also been known to be sex specific. Aromatase activity and expression was found to be greater in female than in male primary cultured cortical astrocytes. Arimidex, an aromatase inhibitor, abolished sex differences in astrocytic cell death induced by oxygen–glucose deprivation (OGD), while addition of estradiol protected both sexes. This implied that local estradiol production by aromatase is protective in both sexes [18]. Studies in aromatase knockout mice (ArKO) further clarified the role of aromatase. Female ArKO mice subjected to reversible middle cerebral artery occlusion had larger infarcts compared to wild type mice. Similarly, wild type female mice treated chronically with the aromatase inhibitor, fadrozole, had more ischemic damage when compared with ovariectomized females, suggesting that extragonadal estradiol is also important for neuroprotection [12]. The role of aromatase in cardiovascular disease has also been highlighted by studies in breast cancer patients who were administered aromatase inhibitors [19]. A recent study demonstrated increased risks of heart failure and cardiovascular mortality in patients taking aromatase inhibitors as compared with patients taking tamoxifen [20]. Administration of the specific aromatase inhibitor, fadrozole, in male rats enhanced the neurodegenerative effects of kainic acid and this was reversed by administration of estradiol, confirming that neuroprotective effects of aromatase are mediated by estradiol [21]. Consistent with these reports in preclinical literature, our clinical study suggests that the aromatization pathway may become active in the event of brain injury, as evidenced by the increased levels of serum aromatase seen after stroke in women. This may be an early endogenous protective mechanism of the brain activated by transcription factors like hypoxia-inducible factor or cytokines [11]. Future studies on the dynamics of these transcription factors, cytokines, and their associations with sex hormone levels may assist in further deciphering the role of these sex hormones in ischemic stroke.

Although no significant difference in estradiol levels was seen after stroke, we found a significant positive correlation between estradiol levels and change in mRS, regardless of sex, which suggests that increased peripheral estradiol level may be associated with a higher mRS, suggestive of worse outcomes. This has been shown in previous studies in elderly women [22–24]. This correlation highlights the complex and long debated role of estradiol in stroke. Boys are known to have higher stroke incidence compared to girls. In fact, it has been seen that for each 1 nmol/l increase in testosterone in young boys, there was a 1.3-fold increase in the risk of stroke [25]. This epidemiology reverses with advanced age, and elderly women have higher stroke incidence and worse outcomes, which is often attributed to the loss of estrogen at menopause. Preclinical studies in stroke models have shown a robust neuroprotective effect of estrogen [5], but this was not recapitulated in early clinical trials. Both the WHI and WEST trials of estrogen supplementation led to increased mortality in post-menopausal women [6, 7], although issues with trial design, including the dose, type, and timing of estrogen supplementation, were raised. The Kronos Early Estrogen Prevention Study (KEEPS) study found that neither oral nor transdermal estrogens affected the progression of atherosclerosis (measured as CIMT, carotid-artery intima–media thickness), when given to recently post-menopausal women [26]. On the other hand, Early versus Late Intervention Trial with Estradiol (ELITE) found that oral estradiol therapy (when initiated within 6 years of menopause) was associated with less progression of subclinical atherosclerosis (measured as CIMT) [27]. It appears that the type, the timing of therapy, and the dose–response effect of estradiol on the vasculature may be important in determining the benefits of exogenous estrogen for stroke prevention. We speculate that higher post-stroke injury (as gauged by worse outcomes, in this case mRS) causes increased aromatase expression and that in turn attempts to increase estradiol levels to protect the brain. However, these results should be interpreted with caution, as causality cannot be determined with a correlation. We did not see any significant difference in estradiol levels with stroke, and there was only an incremental trend. This may be due to a few reasons. It is possible that estradiol levels are not elevated in the hyperacute phase after stroke and increase later than the 24-h time period. This can be ascertained in future studies as we did not perform a longitudinal analysis. Another possible explanation may be that the peripheral endogenous levels of estradiol in the serum are not an accurate representation of the sex steroid milieu in the brain after an injury. Moreover, a low patient sample size to study differences in estradiol levels was one of the limitations of this study.

We also found decreased testosterone levels in men after stroke. This has been reported previously in preclinical models of stroke [28] and also in men [29], which may be due to an acute stress response leading to decreased testosterone levels. However, it is possible that decreasing androgens is a protective mechanism in men. The use of testosterone therapy has been associated with increased risk of adverse cardiovascular outcomes in some studies [30], while others have shown the benefit of testosterone replacement therapy, therefore making the role of testosterone in stroke and cardiovascular disease unclear [31]. Our study did not find any correlation of testosterone levels with mRS, but again, causality is difficult to establish at one time point after stroke.

## Limitations

This study had some limitations which should be considered. Due to the limited number of patients meeting the inclusion and exclusion criteria, our sample size was relatively small, especially for multivariate analysis. Obesity is a known common comorbidity with cerebrovascular diseases, and adipose tissue is a significant source of aromatase [32]. Our database did not record body mass index for most patients, and therefore, obesity was not accounted for in the multivariate regression model comparing hormone levels. In addition, only total hormone levels were measured by ELISA. It would be interesting to see the distribution between free, total, and sex hormone-binding globulin-bound fractions in future studies. Unlike preclinical studies, it is difficult to obtain pre-stroke serum samples in human stroke biorepositories. Therefore, although the control population does make an adequate comparative group, the pre-stroke differences in hormone levels in this patient population could not be ascertained in this study. It should also be realized that the levels of peripheral endogenous hormones may not simulate the endogenous levels of sex steroids in the injured brain. Finally, this study was performed on serum samples at only 24-h time point after stroke. Future studies assessing the hormone levels longitudinally would help us in understanding the complex dynamics of sex steroids after stroke.

## Perspectives and significance

In summary, our study demonstrated an increase in aromatase levels after stroke in post-menopausal women. Aromatization may be a protective mechanism by which estradiol is produced locally in the brain [33]. We speculate that higher injury post-stroke (as determined by higher NIHSS and mRS) accelerates the aromatization process, at least acutely. This is the first study to measure the milieu of sex steroids after stroke in both men and women. This study may add some value in understanding the roles of sex hormones and their contributions to the sexual dimorphism in ischemic stroke.

## Data Availability

All data generated or analyzed during this study are included in this published article.
